# Radiotherapy in metastatic castration resistant prostate cancer patients with oligo-progression during abiraterone-enzalutamide treatment: a mono-institutional experience

**DOI:** 10.1186/s13014-019-1414-x

**Published:** 2019-11-14

**Authors:** Maurizio Valeriani, Luca Marinelli, Serena Macrini, Chiara Reverberi, Anna Maria Aschelter, Vitaliana De Sanctis, Paolo Marchetti, Lidia Tronnolone, Mattia Falchetto Osti

**Affiliations:** 1 Department of Radiation Oncology, “Sapienza” University, Sant’Andrea Hospital, Via di Grottarossa 1035-1039, 00189 Rome, Italy; 2 Department of Oncology, “Sapienza” University, Sant’Andrea Hospital, Rome, Italy

**Keywords:** Oligoprogressive castration resistant prostate cancer, Androgen receptor targeted therapy, Conformal radiotherapy and stereotactic body radiotherapy

## Abstract

**Background:**

Some patients experience oligo-progression during androgen receptor targeted therapy (ARTT) treatments. This progression might not indicate a real systemic drug resistance, but a selective monoclonal resistance. With the aim to delay the start of new line treatments we treated oligo-progressive sites with radiotherapy.

**Methods:**

From June 2011 to Febrary 2019, 29 consecutive metastatic castration resistant prostate cancer (mCRPC) patients were submitted to radiotherapy for oligo-progression (1–3 sites) during ARTT for a total of 37 lesions treated. Thirty-one (83.8%) lesions were treated with conformal radiotherapy and 6 (16.2%) with stereotactic radiotherapy. After radiotherapy all patients continued ARTT.

**Results:**

Median OS (calculated from ARTT start) was 46,6 months (range 4.4–97.5 months), 2 and 3-year OS were 82.8 and 70.7%, respectively. Median PFS was 18,4 months (range 4.4–45.3 months), 2 and 3-year PFS were 38.3 and 8.5%, respectively. Median overall duration of ARTT treatment was 14.8 months (range 4.4–45.3 months) and median duration of ARTT after radiotherapy was 4.6 months (range 1–33.8 months). Patients submitted to radiotherapy > 6 months from the start of ARTT presented a better PFS (*p* < 0.001) and a trend toward a better OS (*p* = 0.101). None patient presented RT and drug related toxicities.

**Conclusions:**

Radiotherapy of oligoprogressive sites may prolong the duration of disease control under ARTT in mCRPC patients with a possible delay in the start of new line treatment. Patients progressing within 6 months from the start of ARTT did not benefit from this approach. More studies are necessary to confirm our results and to evaluate other prognostic factor in order to select patients with high benefit from this approach.

## Background

Advances in clinical and molecular imaging techniques have led to the recognition of an intermediate state of metastatic prostate cancer (mPCa) in which the disease has extended beyond the prostate, although with limited spread to distant organs. This state was first identified and given the terminology, oligometastasis, in 1995 by Hellman and Weichselbaum [1]. Oligometastatic disease can be considered a heterogeneous disease entity with distinct metastatic phenotypes: molecular pathological analysis has suggested that a lethal clone may originate from the metastatic lesion [2]; while further researches have shown that micro-RNAs damage involved take place in distinct regulation processes in oligometastatic vs polymetastatic disease [3]. The clinical implication of this hypothesis is that local consolidative treatments of the primary tumor, metastasis-direct therapy and systemic chemo-hormonal therapy may improve survival for selected patients with mPCa.

There is no consensus on the definition of oligometastatic disease. Some definitions incorporate the site and number of metastases to define the oligometastatic state. In fact, literature suggests reporting five variables in the description of oligometastatic disease: the distinction between synchronous and metachronous metastases, the number and site of lesions, castrate-resistant status, and lastly the imaging modality used to define oligometastatic disease. With the progress of molecular imaging techniques, more and more metastases are being detected. Therefore, many patients considered to be non-metastatic (M0) on conventional imaging might have oligometastatic disease, particularly nowadays as imaging is performed at lower PSA thresholds compared to the past [4–6]. To date, three categories of oligometastatic PCa have been defined: de novo oligometastases (synchronous oligometastases), recurrent (metachronous oligometastases), and progressive (induced oligometastases).

Prostate Cancer Working Group 3 (PCWG3) has defined oligoprogression such as the first evidence of one new lesion or increased volume of one single existing lesion [7]. Treating metastatic prostate cancer patients is an arguing challenge.

Castration resistant prostate cancer patients are a niche of patients affected by progressive prostate cancer despite androgen deprivation therapy and a serum testosterone value < 50 mg/dl. Up to date, EAU-ESTRO guidelines recommend the use of six different agents in this asset of patients: abiraterone acetate plus prednisone, enzalutamide, Ra 223, docetaxel, cabazitaxel and sipuleucel-T. Among all of them, androgen receptor targeted therapy (ARTT) as it has been shown for Abiraterone and Enzalutamide in phase III clinical trials, has high efficacy in patients with metastatic castration resistant prostate cancer (mCRPC), leading to improved overall survival [8, 9].

Abiraterone inhibits intratumoral production of androgens, thereby decreasing engagement of the androgen receptor (AR) for nuclear signaling. Enzalutamide inhibits AR signaling by directly binding to AR. Acquired resistance to both Abiraterone and Enzalutamide inevitably develops due to AR mutations and other escape mechanisms [10].

Some patients treated with ARTT may present an oligoprogression that, probably, does not represent a systemic drug resistance. We hypothesized that the irradiation of lesions progressing on ARTT would likely be effective as resistant lesions are ablated, while continuing ARTT keeps responsive or stable lesions suppressed.

Minding these assumptions, we sought to demonstrate that treating oligo-progressive sites with radiotherapy can prolong ARTT duration. We analysed also overall survival, progression free survival and prognostic factors in order to characterize patients that may beneficiate from this approach.

## Methods

### Patients population

From September 2015 we started to treat mCRPC patients pharmacologically submitted to androgen receptor targeted therapy (ARTT) in Oligoprogression (1–3 new metastasis) with ablative or palliative radiotherapy. Until February 2018, 37 lesions were treated in 29 consecutive cases. ARTT consisted of Abiraterone in 21 cases (72.4%) and Enzalutamide in 8 cases (27.6%).

Median age at ARTT start was 72 years (range 53–91 years) and median time between the onset of androgen deprivation therapy (ADT) and the onset of ARTT was 48.1 months (12.1–123.3 months).

Some patients received previously chemotherapy and most were deemed unfit to chemotherapy due to their physical/clinical conditions, thus they were addressed to ARTT. Seventeen (65.5%) patients received ARTT as first line treatment, 10 (34.5%) as second line (after docetaxel) and only 2 (6.9%) as third line treatment.

### Treatment application and follow-up

Six lesions (16.2%) were treated with streotactic body radiotherapy (SBRT) and 31 (83.8%) with conformal palliative radiotherapy (3DCRT). Lesions with a volume under 15 cm^3^ and, in case of bone lesions, without involvement of posterior wall of vertebral body were treated with SBRT, whereas the others with 3D conformal radiotherapy. The treated lesions were the only apparent site of disease for all patients.

All patients presented rising PSA level during treatment with ARTT and were submitted to F-Choline PET-TC scan that revealed an oligo-progressive pathological uptake. Patients treated with SBRT were also submitted to contrast enhanced magnetic resonance (MR) of the skeletal segment interested. Internal review board approved this study. All patients provided written informed consent. Patient characteristics are summarized in Table 1.

**Table 1:** Patients Characteristics (*n* = 29)

	Details	Patients (%)
Age	Median (range), years	72 (53–91)
Time between ADT and ARTT	Median (range), months	48.1 (12.1–123.3)
RT techniques	3DRT	24 (82.7%)
	SBRT	5 (17.3%)
ARTT	Abiraterone	21 (72.4%)
	Enzalutamide	8 (27.6%)
Treatment line	I	17 (58.6%)
	II-III	12 (41.4%)
PSA after 1 month from the start of ARTT	Median (range), ng/ml	3.02 (0.02–100)
	≤50% reduction	5 (17.2%)
	> 50% reduction	24 (82.8%)
RT timing respect the start of ARTT	< 6 months	15 (51.7%)
	> 6 months	14 (48.3%)
RT sites		Lesions (%)
	Bones	31 (83%)
	Lung	2 (5.5%)
	Lymphnodes	2 (5.5%)
	Prostatic Bed	2 (5.5%)

*ADT* Androgen deprivation therapy, *ARTT* Androgen receptor targeted therapy

*RT* Radiotherapy, *3DRT* Conformal radiotherapy, *SBRT* Stereotactic body

Radiation therapy, *PSA* Prostatic specific antigen

Twenty-two patients (75.9%) received single target treatment, 6 (20.7%) presented 2 lesions and 1 (3.4%) 3 lesions. Thirty-one (83%) out of 37 treated lesions were located in the bone, which represented the majority of the metastatic sites, 2 (5.5%) in lung, 2 (5.5%) in prostatic bed and 2 (5.5%) lymph nodes (lumbar-aortic and internal iliac stations).

All patients underwent a pre-treatment planning CT (2.5 mm slice thickness) in the supine position with feet rests. Planning CT images were fused with MR images and/or F-Choline TC-PET using automatic matching to help gross target volume (GTV) delineation.

For patients treated with SBRT the GTV was expanded of 5 mm in all direction isometricly to obtain PTV. For patients treated with palliative radiotherapy the GTV encompassed the bone or lymph-nodal lesion. Clinical target volume (CTV) encompassed GTV plus 5 mm margin and, Planning Target Volume (PTV) was generated by adding a 5 mm isometric margin to CTV.

Treatment was delivered by a linear accelerator using 6–15 MV photons. Thus, the PTV received 27 Gy in 3 fractions (9 Gy per fraction) in bone lesions treated with SBRT, 54 Gy in 3 fractions (18 Gy for fraction) for lung lesions and 20–30 Gy in 5–10 fractions in patients treated with palliative dosage. The mean value of isodose line covering PTV was 94% (range 90–98%).

After radiation treatment patients continued ARTT with a first PSA assessment after 1 month and then every 3 months. Patients with PSA reduction continued follow up until PSA rising and/or appearance of new symptoms. Then, patients underwent a new F-Choline TC-PET and in case of appearance of new lesions were shifted to other therapy. Toxicities were assessed at each follow up according to the Radiation Therapy Oncology Group (RTOG) scale for acute and late adverse effects [11].

### Statistical analysis

Overall survival (OS) and progression free survival (PFS), were calculated to the event using the Kaplan–Meier method. OS was calculated from the start of ARTT to last follow-up or death. PFS was calculated from the start of ARTT to the evidence of radiological progression that determinated the end of ARTT and the change of treatment. Receiver Operating Characteristic curves were used to find cut-off values for continuous variables. Sub-group analysis was performed stratifying patients treated < 6 month vs >  6 months from the start of ARTT, patients treated with SBRT vs. 3DRT, patients receiving ARTT as first line vs. as second-third line, patients with < 50% PSA reduction vs. > 50% at I follow-up. Statistical analysis was done using SPSS statistical software package version 22.0 (SPSS, Inc., Chicago, IL). A *p*-value lower than 0.05 was considered statistically significant.

## Results

Median follow-up for surviving patients was 36.3 months (range 12.9–68.9 months).

Median overall duration of ARTT treatment was 14.8 months (range 4.4–45.3 months) and median duration of ARTT after radiotherapy was 4.6 months (range 1–33.8 months).

### Progression free survival and prognostic factors analysis

**Fig. 1:**
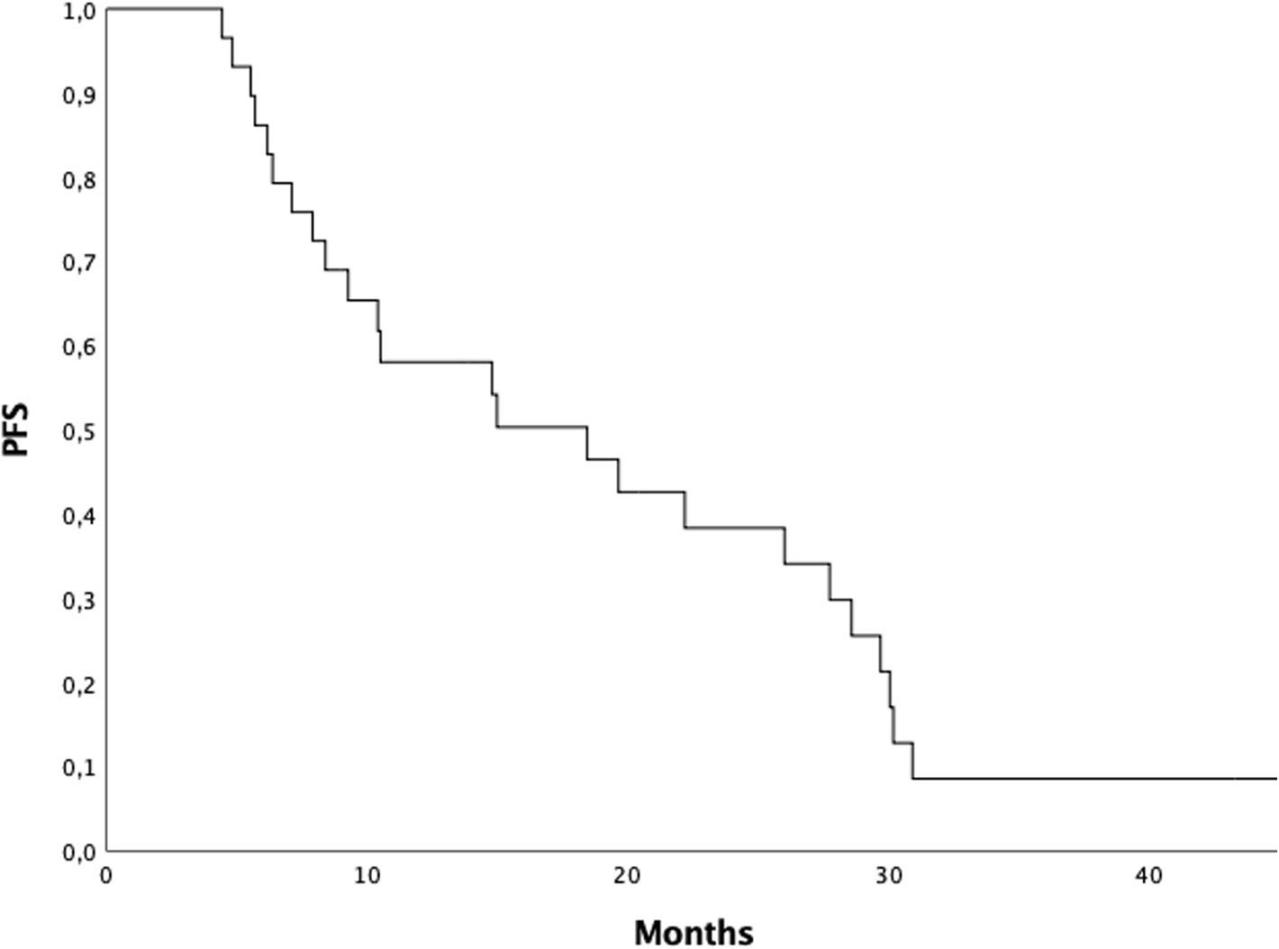
Progression free survival

Twenty-five patients (86.2%) progressed on multiple sites and thus interrupted ARTT and started new treatments; otherwise 4 (13.8%) are still on the same treatment at the end of follow-up. Median PFS was 18,4 months (range 4.4–45.3 months), 2 and 3-year PFS were 38.3 and 8.5%, respectively (Fig. 1).

Median PFS for patients treated with ARTT as first line was 14.9 months (range 4.3–45.3 months) and in patients treated as second-third approach was 14.2 months (range 6.1–43.2 months) (*p* = 0.400).

Patients with < 50% and > 50% PSA reduction 4 weeks after the start of ARTT presented a median PFS of 26.0 months (range 5.5–30.1 months) and 14.2 months (range 4.3–45.3 months), respectively (*p* = 0.872).

Median PFS for patients treated with SBRT was 20.4 months (range 6.3–30 months) and 11.6 months (range 4.3–45.3 months) for those treated with 3DCRT (*p* = 0.765).

**Fig. 2:**
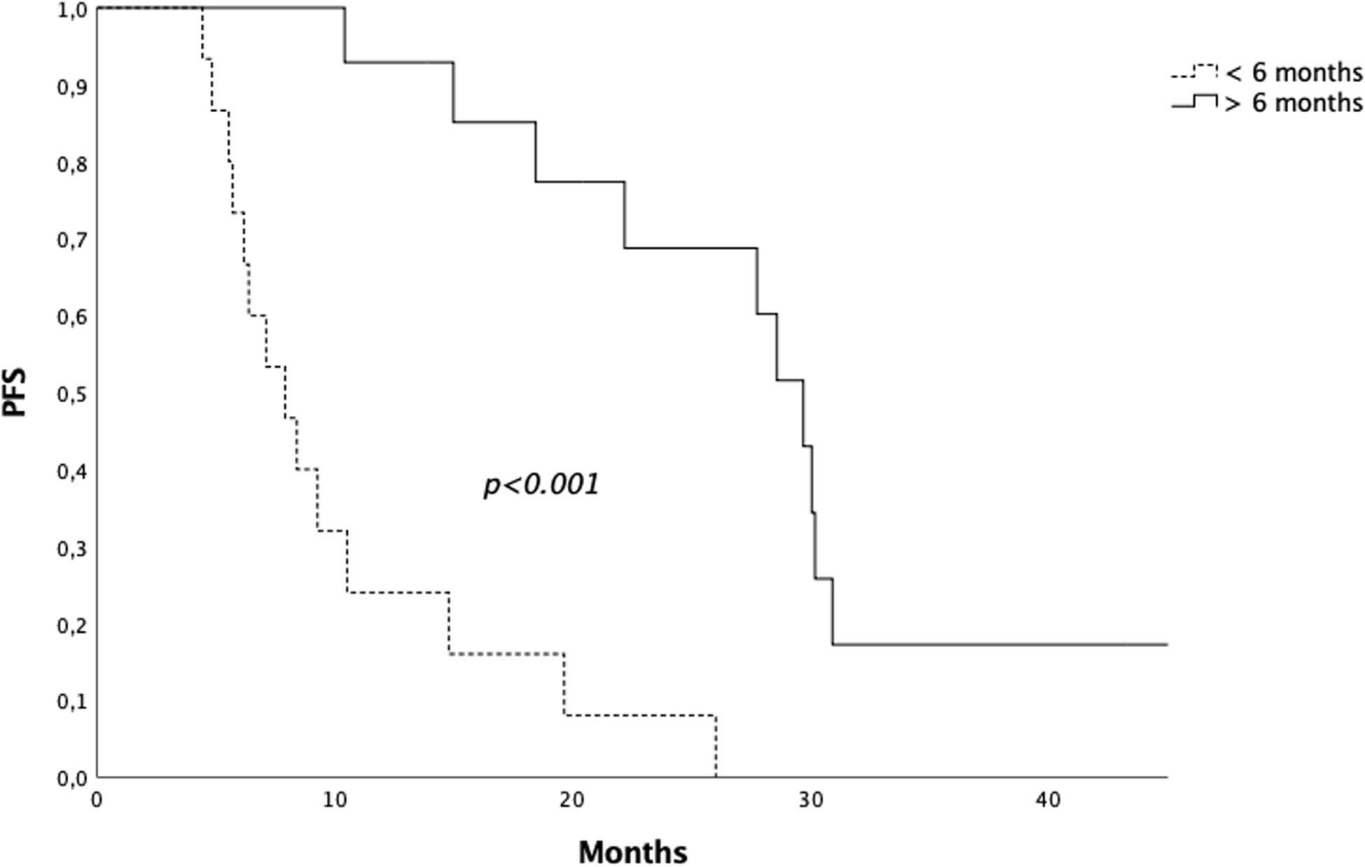
Progression free survival for patients submitted to radiotherapy < 6 vs > 6 months after the start of androgen receptor targeted therapy

Patients submitted to radiotherapy < 6 and >  6 months after the start of ARTT presented a median PFS of 7.9 months (range 4.3–26 months) and 27.7 months (range 10.4–45.3 months), respectively (*p* < 0.001) (Fig. 2).

### Overall survival and prognostic factors analysis

**Fig. 3:**
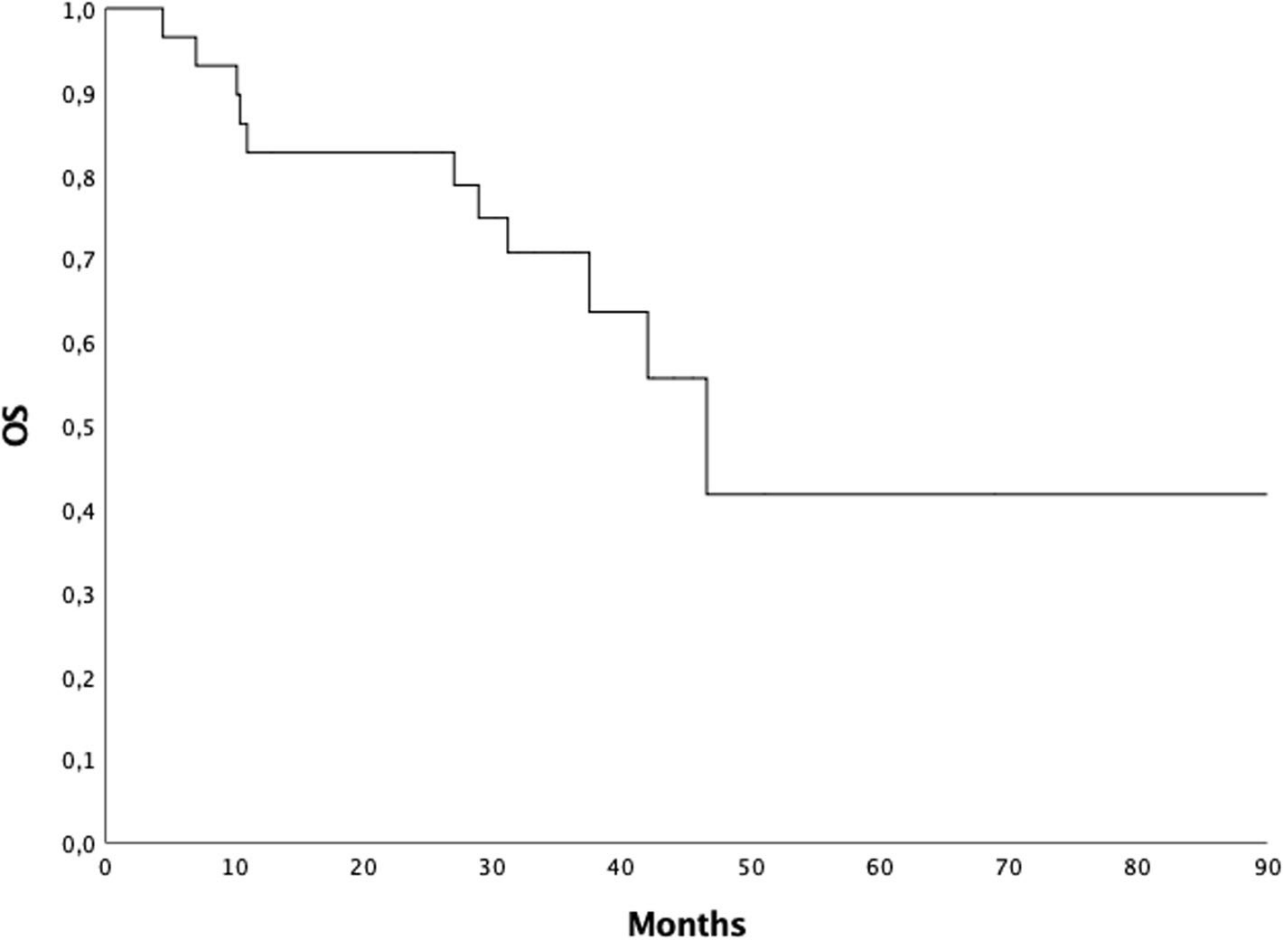
Overall Survival

Median OS was 46,6 months (range 4.4–97.5 months), 2 and 3-year OS were 82.8 and 70.7%, respectively (Fig. 3). At the time of analysis 10 patients (34.5%) had died due to prostate cancer, 15 (51.7%) were alive with disease and 4 (13.8%) were alive without evidence of tumor burden.

Median OS for patients treated with ARTT as first line was 33.2 months (range 4.4–97.5 months) and in patients treated as second-third approach was 36.5 months (range 7–68.9 months) (*p* = 0.583).

Five patients (17.2%) presented with a < 50% PSA reduction after 4 weeks from start of ARTT, 24 (82.8%) > 50%. Median OS for patients with < 50% PSA reduction was 33.2 months (range 28.9–46.5 months) and 35.6 months (range 4.4–97.5 months) for those with > 50% PSA reduction (*p* = 0.553).

Five patients (17.2%) were submitted to SBRT, 24 (82.8%) to 3DCRT. Median OS for patients treated with SBRT was 35.8 months (range 27–45.5 months) and 34.3 months (range 4.4–97.5 months) for those treated with 3DCRT (*p* = 0.502).

**Fig. 4:**
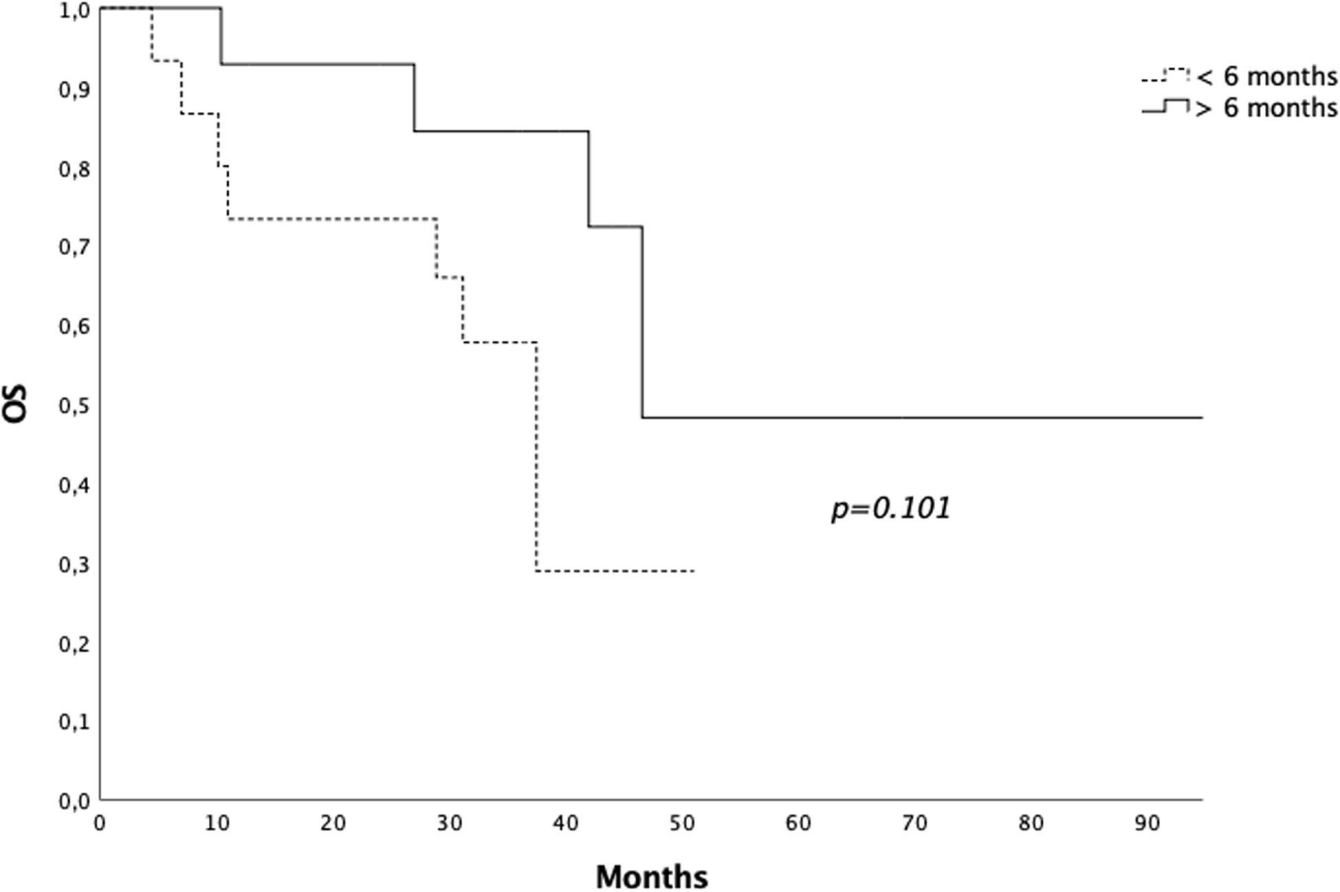
Overall survival for patients submitted to radiotherapy < 6 vs > 6 months after the start of androgen receptor targeted therapy

Fifteen patients (51.7%) underwent radiotherapy before 6 months from the start of ARTT and 14 (48.3%) after. Median OS for the first group was 37.5 months (range 4.4–51 months) and 46.6 months (range 10.4–97.5 months) for the second group (*p* = 0.101) (Fig. 4). Data are summarized in Table 2.

**Table 2:** Univariate analysis

	Median OS (months)	2-year OS (%)	*p*-Value
ARTT line			0.583
I line	33.2	88.2	
II-III line	36.5	75	
PSA reduction			0.553
< 50%	33.2	80	
≥ 50%	35.6	79.2	
RT technique			0.502
SBRT	35.8	80	
3DRT	34.3	79.2	
RT and ARTT			0.101
< 6 months	37.5	73.3	
> 6 months	46.6	92.9	
	Median PFS (months)	2-year PFS (%)	*p*-Value
ARTT line			0.4
I line	14.9	40.3	
II-III line	14.2	32.4	
PSA reduction			0.872
< 50%	26	60	
≥ 50%	14.2	32.9	
RT technique			0.765
SBRT	20.4	60	
3DCRT	11.6	33.9	
RT and ARTT			< 0.001
< 6 months	7.9	8	
> 6 months	27.7	68.8	

*ARTT* Androgen Receptor Targeted Therapy, *RT* Radiotherapy,

*SBRT* Stereotactic Body Radiotherapy, *3DCRT* Conformal radiotherapy,

*OS* Overall Survival, *PFS* Progression Free Survival,

### Pain and toxicity

Before radiotherapy 28/29 patients (96.5%) presented with pain (median Numerical Rating Scale value 7, range 2–9); after radiotherapy 14/29 patients (48.3%) presented pain with a median NRS value of 2 (range 2–4).

During and after the treatment no toxicities were recorded.

## Discussion

Generally, beyond 2 or 3 years from the start of ADT most of patients progress to a castration resistant phase [12], although they still could be androgen dependent. This led to the introduction of new specific drugs such as Abiraterone Acetate and Enzalutamide [13] in the daily clinical practice to lengthen overall survival in this asset of patients.

Factors to consider when describing oligometastatic disease include the distinction of synchronous versus metachronous metastases, the number and site of lesions, the method of imaging, and whether the patient is castration-naive or castration-resistant. Numerous studies have proposed different definitions but the optimal cut-off cannot yet be defined. The term “oligoprogression” indicates a clinical condition in which the systemic disease is still controlled by systemic therapy except for a defined number of sites of relapse. In oligometastatic patients systemic therapy isn’t enough but they may benefit of a local treatment.

Prostate Cancer Working Group 3 (PCWG3) has defined oligoprogression such as the first evidence of one new lesion or increased volume of one single existing lesion. Treating metastatic prostate cancer patients is an ongoing challenge.

The addition of RT to ongoing ARTT has a strong biological rationale. Radiotherapy induces cell death by disrupting various parameters of cell biology necessary for survival, stimulates the dying cells to release a range of molecules (often termed “danger signals”) that in turn could render cancer cells more susceptible to an immune-mediated cytotoxic environment [14–17] and could prevent metastasis to metastasis seeding. In fact, Goundem et al. [18] clinically demonstrated this phenomenon suggesting that the tumour cell populations with a significant survival advantage are not confined within the boundaries of an organ site but can successfully spread to and reseed other sites.

The role and the efficacy of radiotherapy as well of 3DCRT as of SBRT in oligometastatic or oligoprogressive mPCa patients has already been demonstrated in the castration sensitive and castration resistant phase [19].

Tabata et al. [20] investigated bone metastatic prostate cancer patients (mPCa) with < 5 oligometastases treated with median dose of 40Gy. The 3-year OS rates for all patients, for patients that received a dose of ≥40 Gy, and for those that received < 40 Gy were 77, 91, and 50%, respectively. Of note, 87.5% of patients experienced a relief of pain at 1 month, and pathological fracture and spinal cord compression did not occur at the irradiated sites..

Schick et al. [19] treated patients with ≤5 distant and/or regional metastases involving lymph nodes (LN), bone and/or lung lesions detected by 18F-choline 11C-acetate PET-CT. All but one patient were treated with radiotherapy and concurrent ADT. The 3ys BCR-free survival was 55%, the 3ys OS was 92%.

Triggiani et al. [20] treated 86 oligoprogressive castration-resistant prostate (OP-CRPCP) cancer patients with SBRT reporting a 2-year distant progression-free survival of 33.7% and a median systemic treatment-free survival of 21.8 months.

Yoshida S et al. [21] analysed 38 OP-CRPC patients scheduled to receive 60 to 78 Gy (2 Gy per fraction) to the prostate/lymph node metastasis and 30 to 39 Gy (2–3 Gy per fraction) to the bone metastasis. Results show a median time to PSA progression of 8.7 months with a better prognosis for patients having intrapelvic progression disease.

Recently, the concept to continue a therapy beyond progression in case a systemic treatment was effective to control most sites of disease, but one or only a few sites progressed or a few new lesions appeared was developed. In this case, a local therapy such as radiotherapy could possibly control the new sites or the sites of progression maintaining the efficacy of systemic treatment [7]. Thus, this strategy could permit to delay the change of therapeutic strategy.

In our study the median duration of ARTT after radiotherapy was 4.6 months (range 1–33.8 months) and total duration of ARTT was 14.8 months (range 4.4–45.3 months). Detti et al. [22, 23] reported, in a series of 32 patients submitted to radiotherapy for oligoprogression during abiraterone treatment, a median abiraterone duration of 13.0 months (range = 3.8–40.9 months) and median duration of abiraterone after RT of 7.2 months (range = 0.1–29.7 months).

In our study we treated patients that presented a minimum of 12 months interval between the start of androgen deprivation therapy (ADT) and the progression to a castration resistant phase and then, probably, with longer survival rates despite the metastatic progression. The duration of hormonal-sensitive phase is a known prognostic factor. In fact the analysis of the control arm (patients submitted to ADT only) of the STAMPEDE trial [24] demonstrated a correlation between Failure Free Event survival (FFS) and Overall Survival (OS); in this study as longer was the FFS as long was OS.

Moreover, we used SBRT in only 5 patients (6 lesions treated) whereas the majority of patients received a 3DCRT with palliative dosage (30 Gy in 10 fractions or 20 Gy in 5 fractions) due to volume or site of the lesions. It is irrefutable that SBRT, when feasible, is the standard for treatment of oligorecurrent or oligoprogressive lesions [25, 26], but, in our opinion, 3DCRT might represent a valid alternative in cases not candidate to SBRT, as demonstrated also from the study of Detti et al. [23].

We analysed prognostic factor such as type of treatment (SBRT vs. 3DCRT), ARTT as first approach vs. II-III approach, < 50% vs. > 50% PSA reduction after 4 weeks from the start of ARTT without any significant difference in term of OS and PFS. These results could be due to a selection bias since only 5 patients underwent SBRT and presented < 50% PSA reduction after ARTT. Instead, there was a trend toward a better OS in patients that received radiotherapy 6 months after the start of ARTT vs. before 6 months (median OS 46.6 months vs. 37.5 months; *p* = 0.101) and a significant difference in term of PFS (median PFS 27.7 months vs 7.9 months; *p* < 0.001). Thus patients that progressed within 6 months from the start of ARTT did not benefit from an intensification of treatment. Analysing data from COU-AA 302 and PREVAIL [27] the median radiological progression free survival (rPFS) in the control groups were 8.3 and 3.9 months, respectively. This could signify that patients under ARTT that progressed between 4 and 8 months (in our case 6 months) from the onset of treatment have behaviour similar to patients that did not receive ARTT.

A recent consensus statement of Italian Association of Radiotherapy and Clinical Oncology (AIRO) [28] confirm our hypotesis on the role of local treatment, such as radiotherapy, to sites of progressive disease as alternative to systemic treatment shift, in asymptomatic or minimally symptomatic oligoprogressive CRPC patients.

Limitations of our study are: low number of patients enrolled, retrospective evaluation and heterogeneity of radiotherapy schedule used.

With these limitations, our study demonstrated that metastasis directed radiotherapy in oligoprogressive mCRPC patients treated with ARTT delay the start of new treatment line for a median time of 4.6 months, other than relieve pain in most of them. The rationale of delay new treatment line start is to lengthen OS in patients with a slowly progressive disease (as demonstrated by the minimum interval of 12 months from the beginning of ADT and the onset of castration resistance phase).

Further studies are necessary to confirm or not the results of current study and to search other prognostic factor in order to select oligoprogressive patients that might have an advantage from a local treatment without interruption of ARTT in respect to those that need an immediate change of therapeutic strategy.

## Conclusion

Radiotherapy of oligoprogressive sites may prolong the duration of disease control under ARTT in mCRPC patients with a possible delay in the start of new line treatment. Patients that progressed within 6 months after the start of ARTT did no benefit from this approach. Prospective randomized studies and studies with more cases are necessary to confirm our results and to evaluate other prognostic factor in order to select patients with a high benefit from this approach.

## Data Availability

The datasets used and/or analyzed during the current study are available from the corresponding author on reasonable request.
